# Evaluation of the predominant bacteria and proinflammatorycytokine expressions in odontogenic cysts: Predominant bacteria in odontogenic cysts

**DOI:** 10.3724/abbs.2022001

**Published:** 2022-01-29

**Authors:** Yan Li, Qingran Yang, Chi Yang, Kan Ding, Guangzhou Xu

**Affiliations:** 1 National Clinical Research Center for Oral Diseases Shanghai Key Laboratory of Stomatology&Shanghai Research Institute of Stomatology Department of Oral Surgery Shanghai Ninth People’s Hospital College of Stomatology Shanghai Jiao Tong University School of Medicine Shanghai 200011 China; 2 Glycochemistry and Glycobiology Lab Shanghai Institute of Materia Medica Chinese Academy of Sciences Shanghai 201203 China; 3 Zhongshan Institute for Drug Discovery Shanghai Institute of Materia Medica Chinese Academy of Sciences Zhongshan 528400 China

Odontogenic cysts (ODCs) originated from odontogenic epithelium are the most common cyst that may exhibit local destructive behavior. Radicular cysts (RCs) and developmental odontogenic keratocysts (OKCs) are the two most commonly encountered types in practice. Infection of necrotic teeth is responsible for the formation of RCs and progresses as apical lesions, whereas OKCs react with an inflammatory response to chronic irritation caused by necrotic teeth [1]. Cystic lesions may lead to the loosening of the teeth, predisposition to fracture of the involved bone, inflammatory consequences, pain, swelling, andother symptoms.

Through the years, there have been conflicting reports regarding the presence of microorganisms in odontogenic cysts. Some previous studies demonstrated that bacteria were not found in odontogenic cysts and did not normally penetrate into the lamina, showing no aerobe and anaerobe bacteria with microbiologically sterile cystic fluid [ 2, 3]. However, other studies revealed that bacteria acted synergistically to produce pathology and fluids of the odontogenic cysts, suggesting that proinflammatory cytokines and inflammation-associated growth factors may play a role in the development of cysts [ 4, 5]. For example, IL-1α and TNF-α are important mediators contributing to the development of cysts and to enhance the activity of osteoclasts’ bone resorption [5]. However, few studies have been carried out to investigate the levels of cytokines in ODCs. The aim of this study is to define the responsible bacteria of ODCs and determine the levels of macrophage-originated cytokines in response to bacterial infection in ODCs fluids.

Data used in the present study were collected at the Department of Oral Surgery, Shanghai Jiao Tong University. The study population consisted of systemically healthy patients having ODCs with jaw expansions, along with a history of infection and purulent cyst fluid. Only the expansive cysts were chosen because of the easiness in obtaining the cyst fluids. CT scans were used to evaluate the expansion size of the buccal and/or lingual bony corteces. The patients who were under antimicrobial therapy and were receiving antiviral or immunosuppressive therapies, and the patients with an obvious mucosal breach or portal entry for infection via the oral cavity were excluded from this study. The patients who had odontogenic cysts with a diameter of less than 1 cm in size (calculated via the orthopantomographs) were also excluded from this study because of the inability to obtain adequate cyst fluid. A total of 35 patients were enrolled in the study. The study group consisted of males and females whose ages ranged from 10 to 55 years. The average age of all patients included in the study was 32.1±11.3 years; the average age of males was 33.6±11.9 years, and the average age of females was 29.5±10.8 years. The Ethical Committee permission was obtained prior to the commencement of the study.

The clinical materials of 35 patients with cysts were obtained, cultured and then analyzed microbiologically. Out of the 35 enucleated lesions, 18 samples were OKCs, 11 of them were RCs,0 of them were DCs ( Table 1).

**Table:** **Table1** Bacteria isolated from odontogenic cysts

Species of bacteria	OKC	RC	DC	Gram stain
*Staphylococcus warner*	1	1	–	Gram (+)
*Streptococcus dentisani*	1	–	–	Gram (+)
*Streptococcus constellatus*	1	1	–	Gram (+)
*Streptococcus anginosus*	4	3	–	Gram (+)
*Pseudomonas*	11	6	–	Gram (−)

The species of bacteria in these odontogenic cysts were identified by 16S rRNA sequence analysis. The cyst fluids were isolated from odontogenic cysts, followed by strain identification using DNA-based techniques.

The PCR products of the 16S rRNA gene were eluted from an agarose gel using the Gene JET™ Extraction kit (Fermentas, Heidelberg, Germany), and the purified PCR products were sequenced commercially by Sangon Biotech (Shanghai, China). The sequence data were assembled and analyzed using the Lasergene sequence analysis software package (DNA Star, Inc., Madison, USA). Sequence similarity searches using the Basic Local Alignment Search Tool (BLAST) were performed by comparing the sequences obtained with other microbial sequences in the National Center for Biotechnology Information (NCBI) database ( http://www.ncbi.nlm.nih.gov/BLAST/). The 16S rRNA gene sequences of type strains and other strains closely related to our 28 isolates were obtained from the NCBI database.

In 29 (83%) of the studied specimens, five different types of microorganisms were isolated ( Table 1). *Pseudomonas* was the most common type in all samples ( *n*=17; 58.7%). Additionally, *Staphylococcus warner* ( *n*=2; 6.9%), *Streptococcus dentisani* ( *n*=1; 3.4%), *Streptococcus constellatus* ( *n*=2; 6.9%), and *Streptococcus anginosus* ( *n*=7; 24.1%) were identified.

**Figure 1:**
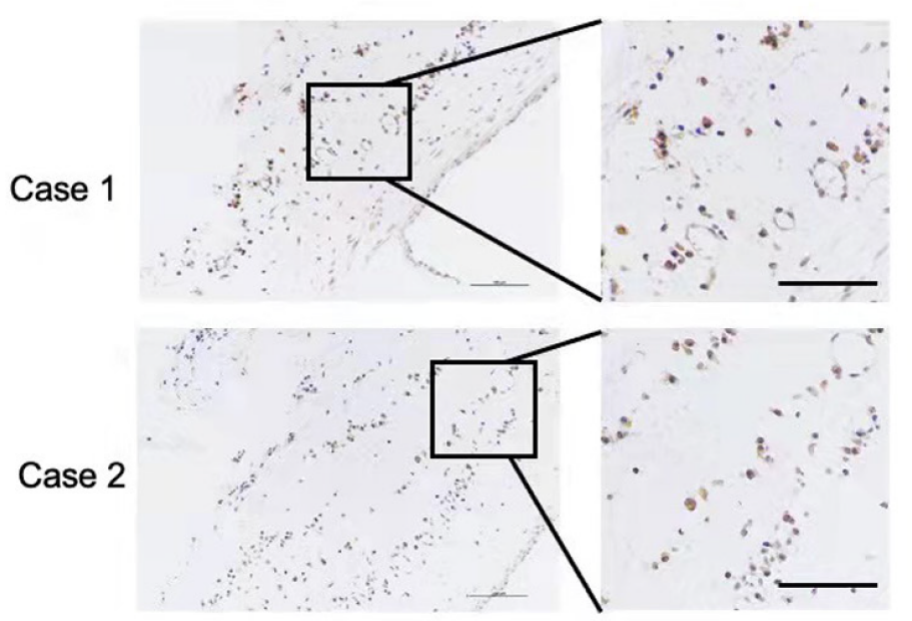
Figure1 **Representative IHC images of cyst tissue detected using an anti-pseudomonas antibody**Scale bar: 100 μm.

About 64.7% of the *Pseudomonas* positive cyst fluids were in the OKC group. The remaining material (35.3%) were in RCs. No bacteria were detected in 6 subjects (17% in total). The status of isolated bacteria from the cultures are shown according to the properties of the cyst fluid ( Table 2). After surgical treatment, the cyst mucosa were obtained from all patients diagnosed with odontogenic cysts clinically and radiologically, and then histopathologically examined to detect pseudomonas using anti- *Pseudomonas*antibody (ab68538; Abcam, Waltham, USA). Immunohistochemistry (IHC) staining of histological sections confirmed the presence of *Pseudomonas* at the tissue level ( Figure 1).

**Table:** **Table2** The relationship between the bacterial growth and properties of cyst fluids

Properties of cyst fluid	Bacterial growth		Case number
	Positive	Negative	
Straw colored fluid	15	5	20
Brownish colored fluid	8	2	10
Straw colored viscous fluid	5	–	5
Case number	28	7	35

IL-1α and TNF-α are important mediators contributing to the development of cysts and the enhancement of the osteoclasts’ bone resorption activity [5]. Therefore, TNF-α and IL-1α in response to bacterial infections were analyzed in ODC lesion. TNF-α and IL-1α protein levels were determined by enzyme-linked immunosorbent assay (ELISA).

**Figure 2:**
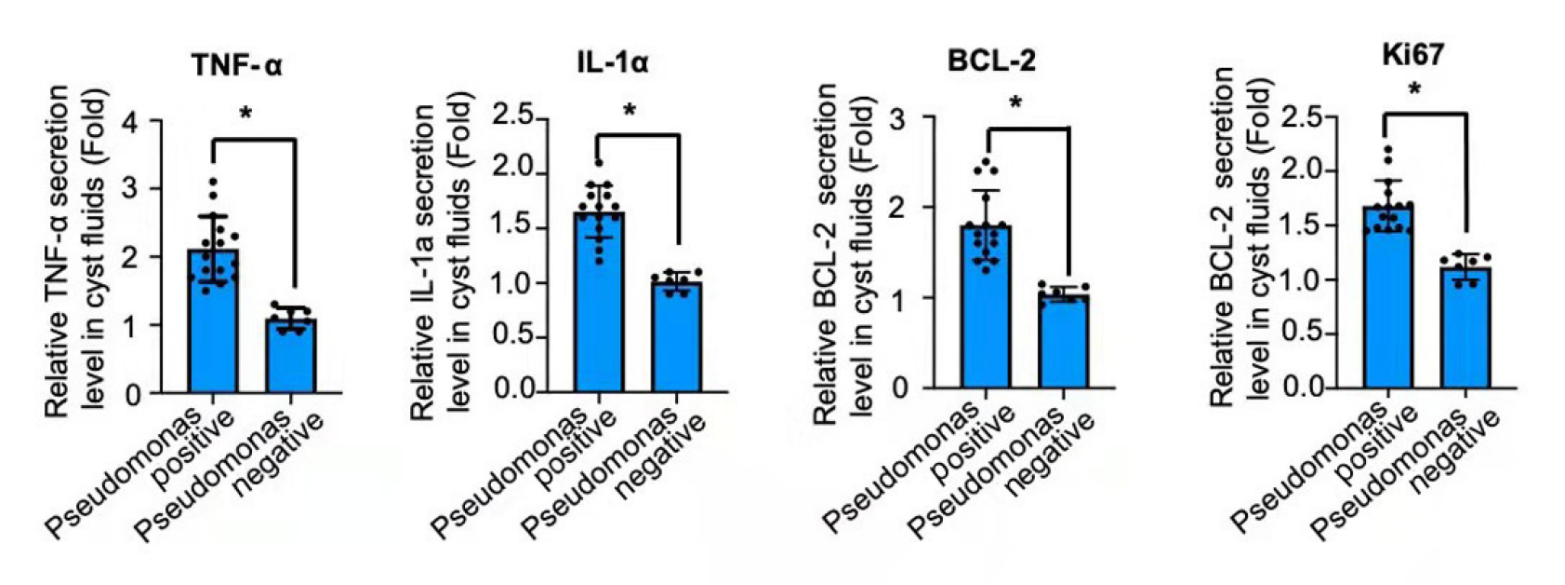
Figure2 **Relative protein levels of secreted factors in the cyst fluids**The secreted factors (TNF-α, IL-1, BCL-2 and Ki67) were determined by ELISA. *P<0.01.

There was a marked difference at the secretion levels of the proinflammatory markers among the groups ( Figure 2). The presence of significantly higher levels of TNF-α and IL-1α in *Pseudomonas*positive fluids and bacterial negative fluids was observed. In addition, *Pseudomonas* positive cyst had about 2-fold increase in these makers over *Streptococcus milleri*group (SMG)-positive cyst, including *Streptococcus constellatus* and *Streptococcus anginosus*.

In addition, BCl-2 and Ki-67 proteins, which are predictive indicators closely associated with cell proliferation, were analyzed in cyst fluids. There was a significant difference in the secretion levels of the proliferation markers among the groups ( Figure 2). The presence of significantly higher levels of BCl-2 and Ki-67 in *Pseudomonas* positive fluids than in bacterial negative fluids were observed. In addition, *Pseudomonas* positive cyst had about 2-fold increase in the makers over SMG positive cyst.

Furthermore, positive bacteria were determined in 83% of the specimens. SMG bacteria are important pathogenic microorganisms for infections and abscess of the orofacial region, according to literature survey. Here SMG ( *S*. *milleri*group positive sample/total bacteria positive samples=31.0%) were isolated from sample cultures. In addition, *Pseudomonas* were found to be higher (58.7%) in bacteria and were predominantly isolated from cultures. *Pseudomonas* are gram negative and facultative anaerobic branched bacteria that are difficult to grow in culture. The pathogenic *Pseudomonas* are considered to be part of normal commensals of the human oral cavity [6]. However, *Pseudomonas* bacteria were not isolated in previous studies. TNF-α and IL-1α are thought to play a role in bone remodeling, bone resorption, and new bone deposition. In this study, the expressions of TNF-α and IL-1α were determined. There are significant differences in the cytokine level among the cyst groups. ELISA results showed that IL-1α and TNF-α were high in *Pseudomonas*-positive cyst fluids, suggesting that the proinflammatory response created by macrophages might be induced in response to *Pseudomonas* bacteria.

Cyst formation, like the development of neoplasms, involves a dysregulation of balance between cell proliferation and cell death [7]. The apoptosis of the cells is largely determined by the interactions among the BCL-2 family members [8]. Most studies on cell proliferation are based on Ki-67 marker. In this study, higher expression levels of BCL-2 and Ki-67 were found in the bacteria-positive cyst fluids, consistent with previous reports [ 9, 10]. In addition, the protein levels of BCL-2 and Ki-67 were the highest in *Pseudomonas* positive cyst fluids. According to our results, *Pseudomonas* exacerbates the pathogenesis by increasing the productions of TNF-α, IL-1α, BCL-2 and Ki-67, suggesting that this facultative anaerobic bacterium may trigger the pathogenesis of cyst formation. However, further studies utilizing a larger sample size and more advanced tools are recommended, and further *in vitro* studies are also required to clarify the role of this bacterium and other cytokines in the pathogenesis of the ODCs.
